# Association of maternal depression and hypothyroidism with infant gastroschisis: a population-based cohort study in Canada

**DOI:** 10.1038/s41598-023-34090-2

**Published:** 2023-05-09

**Authors:** Shiliang Liu, Hughes Claude, Shin Jie Yong, Dunjin Chen

**Affiliations:** 1 Centre for Surveillance and Applied Research, Public Health Agency of Canada, Ottawa, Ontario Canada; 2 School of Epidemiology and Public Health, Faculty of Medicine, University of Ottawa, Ottawa, Ontario Canada; 3 Reproductive Health Center of Excellence and Therapeutic Science and Strategy Unit, IQVIA & Department of OB-GYN, Duke University Medical Center, Durham, NC USA; 4 Department of Biological Sciences, School of Medical and Life Sciences, Sunway University, Selangor, Malaysia; 5 Department of Obstetrics and Gynecology, Third Affiliated Hospital of Guangzhou Medical University, Guangzhou, China

**Keywords:** Developmental biology, Ecology, Ecology, Planetary science, Diseases, Endocrinology, Medical research, Risk factors

## Abstract

Gastroschisis has increased globally over recent decades, and this increase has not been explained by identified risk factors. We conducted a population-based study of infants born in Canada, 2004–2020. We used “*winter*” months (i.e., September through June) and northern areas of residence as indicators of less sunlight/less active lifestyle, while *“summer”* (i.e., July and August) and southern areas were considered as reference. Rate of gastroschisis for infants conceived in *winter* (3.4 per 10,000) was higher than for infants conceived in *summer* (2.2 per 10,000; *p* < 0.001). Exposure to *winter*, and northern area, hypothyroidism, substance or tobacco uses and depressive disorder were initially identified as risk factors for gastroschisis. There was a significant interaction between women < 24 years of age and 2-month conception intervals (rate ratio (RR): 1.42 (95% confidence interval [CI] 1.19–1.70). The association of maternal depression (mean ratio 2.19, 95% CI 0.87–3.50, *p* = 0.001) with infant gastroschisis was mediated by hypothyroidism (mean ratio 1.04, 95% CI 1.01–1.07, *p* < 0.001), whereas substance use, hypothyroidism, tobacco smoking and gestational diabetes showed 5.5-, 3.1-, 2.7-, and 1.2-fold associations, respectively, with maternal depression. In contrast to the *summer* conception interval of low gastroschisis risk, an elevated risk of gastroschisis spans the other ten months in association with higher levels of stress adaptation, thermoregulation and metabolism, reproduction, and growth effector hormones. Our findings suggest that periconception depression with mediation by hypothyroidism, may play a causal role in offspring gastroschisis.

## Introduction

Gastroschisis is a serious defect with midgut prolapse into the amniotic cavity^1^. Its prevalence at birth varies greatly by geographic region^2,3^. Gastroschisis has increased globally over recent decades, and this increase is not explained by demographic changes in maternal age or other identified risk factors. The cause is believed to be multifactorial and primarily non-genetic/epigenetic, but its etiology remains unknown^3–6^. Lifestyle behaviours, environmental exposures, substance or alcohol use, tobacco smoking, poor nutrition, lower dietary intake of vitamin D and sociodemographic risk factors have been frequently or occasionally associated with an increase in gastroschisis^6–8^, young maternal age is the only confirmed risk factor, while the etiology remains obscure. For example, a 7 times higher risk of gastroschisis is typically observed in women < 20 years of age than women aged 25–29 years. Our previous study reported a significantly increasing temporal trend in the maternal age-adjusted rate, and identified maternal hypothyroidism, substance use and northern residence as significant risk factors for gastroschisis in Canada^9^. A recent study of global gastroschisis patterns among 24 countries showed an extremely wide range in the prevalence for teen women (< 20 years): from 2.1 in Slovak Republic or 3.7 per 10,000 births in Spain to 21.5 in Utah (U.S) and 26.2 per 10,000 in Canada^3^. However, researchers have paid little attention to why gastroschisis varies with location (e.g., northern area) of residence and the complex relationship and interplay between maternal characteristics, conditions and identified risk factors, as well as the mechanistic pathway.

We have assumed that circannual endocrine function, photoperiod, residential latitude and climate may influence the relationship between anterior pituitary hormones plus their respective effector hormones and risk of offspring gastroschisis^10–14^. We have used “*winter*” months (i.e., September through June) and northern geographic latitudes as proxy indicators of shorter photoperiod/less outdoor lifestyle exposure, while “*summer*” (July and August) and southern latitudes were considered as the reference exposures that may influence fetal gastroschisis development. As gastroschisis develops between the 5th and 10th week of gestation, a negative relationship with pre- or perinatal thyroid function (circulating hormones) and vitamin D status in winter versus summer season is plausible^8,11,15–17^. Tendler et al. analyzed an Israeli medical record of 46 million person-years, and they found that within the normal ranges of each respective hormone, levels of most anterior pituitary hormones were relatively higher in calendar summer months, while their target-tissue effector hormones were relatively higher across the calendar fall, winter and spring months. Specifically, the study reported that thyrotropins (TSH) increased after May, and peaked in August, and the seasonality increased with latitude^12^. Fu et al. conducted a study among women of reproductive age in Shenyang, northern China and found that central sensitivity to thyroid hormones was influenced by seasonal factors and age^18^. Studies on residents in the Antarctica suggested that prolonged cold exposure was associated with increased TSH and T3 levels during winter compared to summer, and increased sunlight and vitamin D_3_ exposure in summer was found to be positively associated with steroid hormone production of sex hormones^19^.

We examined whether fetuses conceived in *winter* season or in residential areas with high latitudes (shorter photoperiod along with cold climate) have an increased risk of gastroschisis compared to *summer* or southern areas in Canada. Specifically, we intended to assess how extrinsic (e.g., substance use, tobacco smoking) and intrinsic factors (e.g., maternal age, depression, hypothyroidism, gestational diabetes and chronic illness) interacted with conception seasonal variation in infant gastroschisis prevalence, in an attempt to explore the etiological role and possible underlying pathway in fetal gastroschisis development.

## Methods

### Study design and population

We completed a retrospective population-based cohort study. We included all live births (N = 4 435,732) in Canada between April 1, 2004 and March 31, 2020. The data were drawn from the Canadian Institute for Health Information’s Discharge Abstract Database (DAD), which contains records of live-born infants linked to their mothers^20^. The records were collated by trained medical record personnel using standardised definitions and included information on gestational age, plurality, birthweight, maternal and newborn diagnoses (up to 25 diagnosis fields available), and interventions (up to 20 procedure fields) that were noted during the delivery admission^21^. Diagnoses among infants and mothers were coded using the enhanced Canadian version of the 10th revision of the International Classification of Diseases (ICD‐10 CA). The hospital discharge database has been routinely checked for accuracy, and validation studies have shown the information to be complete and accurate^20,22^. Information on pregnancy conditions and birth outcomes was well recorded in the database, and the database has been previously used for perinatal research^9,23–25^.

**Figure 1:**
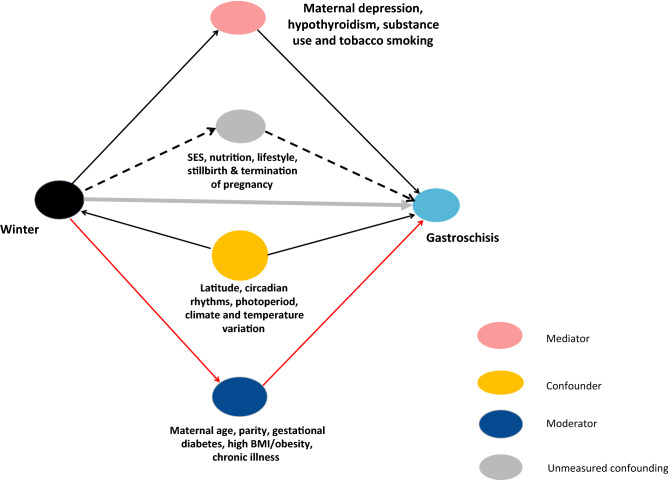
Directed Acyclic Graph. The directed acyclic graph (DAG) above represents associations between covariates (including measured and unmeasured and the primary exposure (winter months) and outcome (infant gastroschisis).

Stillbirths and terminated pregnancies were not included as variables such as gestational age and birth date for fetal deaths necessary to determine conception date were unavailable in the DAD for privacy concern. Information on gestational age at birth in the DAD allows for accurate estimation of conception date for live-born infants^26^. After exclusions for gestational age < 22 weeks, birth weight < 500 g, missing information on birth date, gestational age, birth weight, non-Canadian residence or maternal age at conception ≥ 45 years (n = 26,016); a total of 4,409,716 (99.4%) mother-liveborn dyads remained eligible for the study [s-Fig. 1].

### Conception season (exposure)

Month of conception and maternal residential latitude estimate were used as proxies for exposure to photoperiod and climate variation around the time of conception and in early fetal development. The month of conception, a primary exposure measure, was determined using the birth month and the number of completed weeks of gestation at birth, using the following formula:

$${\text{Month}}\;{\text{of}}\;{\text{conception}} = {\text{birth month}}{-}\left( {\left( {\left( {{\text{gestational}}\;{\text{weeks}}\; \times \;{7}} \right){-}\left( {{14} + x} \right)} \right)/{3}0.{4}} \right),$$

where (1) 7 is used to convert gestational weeks to days; (2) 14 + *x* indicates two weeks (i.e., 14 days) of the last menstrual period (LMP) and *x* represents the missing incomplete gestational week ranging from 1 to 6 days, and (3) 30.4 (i.e., 365 days divided by 12 months) is used to convert gestational days to define conception months such as January, February …… and December.

Pre-defined sub-regions based on postal codes of maternal residence were used to classify pregnant women into four geographic regions with differential latitudes reflecting degree of daylight exposure:^27,28^ (i) ***Far North*** consisting of the three Territories: Yukon, Northwest Territories and Nunavut; (ii) ***North*** including northern British Columbia, Alberta, Saskatchewan and Manitoba, northern Ontario, northern Quebec (iii) ***North-to-South*** consisting of other parts of British Columbia, Alberta, Saskatchewan and Manitoba, and Labrador; and (iv) ***South*** including western, central, and eastern Ontario, metropolitan Toronto, New Brunswick, Nova Scotia, Prince Edward Island, Newfoundland and southern Quebec.

In this study we used *winter* months (i.e., September through June) and northern areas of residence as indicators of less sunlight/less active lifestyle, while *summer* (i.e., July and August) and southern areas were considered as the reference.

### Outcome and covariates

Gastroschisis was collected from the birth record using ICD-10 code (s-Table 1). Gastroschisis is usually detected prenatally and confirmed at birth. In this study, we used hypothyroidism to be a surrogate variable for maternal thyroid dysfunction.

**Table 1:** Maternal and infant characteristics among 4,409,716 Births in Canada, 2004–2020 in Canada, by conception season as specified.

Characteristic	Conception in *Winter* (Exposed) (n = 3689 855, 83.7%)	Conception in *Summer* (Un-exposed) (n = 719 861, 16.3%)	p value for difference ^b^
Period of birth			< 0.001
2004–2007	866,540 (23.5)	170,620 (23.7)	
2008–2011	941,343 (25.5)	184,735 (25.6)	
2012–2015	946,557 (25.7)	183,288 (25.5)	
2016–2019	935,415 (25.3)	181,218 (25.2)	
Geographic region			< 0.001
Far North	43,630 (1.2)	8537 (1.2)	
North	209,264 (5.7)	41,134 (5.7)	
North-to-South	1,503,553 (40.7)	296,323 (41.2)	
South	1,933,408 (52.4)	373,867 (51.9)	
Maternal age at conception (year)			< 0.01
13–18	134,246 (3.6)	26,690 (3.7)	
19–23	5,16,704 (14.0)	100,343 (13.9)	
24–28	1,056,251 (28.6)	207,029 (28.8)	
29–33	1,235,325 (33.5)	240,633 (33.4)	
34–44	747,329 (20.3)	145,166 (20.2)	
Residence			
Rural	619,244 (16.8)	122,112 (17.0)	< 0.001
Urban	3,070,611 (83.2)	597,749 (81.7)	
Parity			< 0.0001
1st child	1,280,636 (34.7)	248,368 (34.5)	
2nd child	1,008,019 (27.3)	198,754 (27.6)	
≥ 3rd child	638 675 (17.3)	123,629 (17.2)	
Missing	762,525 (20.7)	149,110 (20.7)	
Multifetal pregnancy			
Yes	52,985 (1.4)	9908 (1.4)	< 0.001
No	3,636,870 (98.6)	709, 953 (98.6)	
Substance use			
Yes	37,669 (1.0)	6758 (0.9)	< 0.001
No	365,186 (99.0)	713,103 (99.1)	
Depressive disorder			
Yes	11,833 (0.3)	2402 (0.3)	0.08
No	3,678,022 (99.7)	717,459 (99.7)	
Hypothyroidism			
Yes	26,719 (0.7)	5188 (0.7)	0.75
No	3,663,136 (99.3)	566,953 (99.4)	
Gestational diabetes			
Yes	240,089 (6.5)	45,886 (6.4)	< 0.001
No	3,449,766 (93.5)	6,73,975 (93.6)	
Tobacco smoking			
Yes	19,092 (0.5)	3742 (0.5)	0.80
No	3,170,763 (99.5)	716,119 (99.5)	
Obesity			
Yes	59,552 (1.6)	11,790 (1.6)	0.14
No	3,630,303 (98.4)	70,807 (98.4)	
Chronic illness^a^			
Yes	10,750 (1.5)	55,637 (1.5)	0.36
No	3,634,211 (98.5)	368,868 (98.5)	
Infant sex			
Male	1,892,351 (51.3)	368,869 (51.2)	0.50
Female	1,797,504 (48.7)	350,993 (48.8)	

^a^Including pre-gestational type 1 or type 2 diabetes, specified mental disorders, migraine, lupus, epilepsy, hyperthyroidism or thyroiditis among mothers.

^b^*P* values for difference between exposed and un-exposed groups were calculated using χ^2^ test.

Maternal characteristics and covariates studied included maternal age at conception derived using gestational age and date of delivery, depressive disorder, hypothyroidism, use of substances (i.e., opioids, cannabinoids, cocaine, alcohol and other specified/unspecified drugs or medications), tobacco use, gestational diabetes, obesity, and maternal chronic conditions or illnesses (e.g., pre-gestational type 1 or type 2 diabetes, anxiety and other specific mental disorders, migraine, lupus, epilepsy, hyperthyroidism or thyroiditis) (s-Table 1). Other characteristics and covariates included parity (i.e., 1st child, 2nd child,  ≥ 3rd child and missing), multifetal pregnancy and infant sex. Rural or urban maternal residence was identified using the forward sortation area of residential postal code. We included this variable (i.e., rural vs. urban) to potentially account for variations in accessibility to prenatal screening/diagnosis and subsequent termination, and because this variable is a partial surrogate for socioeconomic status (SES)^28^. A directed acyclic graph (DAG) was used to illustrate our data analysis and hypothetical relationships between risk factor, covariates and outcome in this study (Fig. 1).

### Statistical analysis

We examined gastroschisis prevalence in each month of conception. We used July and August (*summer*), months with the longest daytime non-exposure, as the reference. We compared individual 2-month *winter* seasons of exposure period, such as January–February, March–April, May–June, September–October and November–December, and then compared exposure season combined (i.e., year-around) to the reference. Maternal age at conception was grouped as < 19, 19–23, 24–28, 29–33, 34–38 and 39–44 years.

A log-binomial regression model with a Poisson distribution was used to model the association of infant gastroschisis between exposure to conception season and covariates. Univariable and multivariable rate ratios with 95% confidence intervals were estimated. Specifically, interactions between season of exposure (i.e., 2-month conception season) and maternal age at conception (< 24 vs. 24–44 years) were assessed, adjusting for residential latitude, hypothyroidism, substance use, gestational diabetes and other covariates. We compared five two-month *winter* (exposure) conception seasons with *summer* (non-exposure) season, respectively, to assess if there were associations and interactions between maternal age, characteristics, covariates and conception season in infant gastroschisis.

Next, in order to understand the relationships of various risk or “protective” factors/covariates that may be involved in the causal pathway between the hypothesized exposure and risk of offspring gastroschisis, we conducted mediation analysis^29,30^. Specifically, we focused on the assessments of hypothyroidism as a primary mediator for substance use, tobacco smoking, depressive disorder, gestational diabetes and obesity in univariate and multivariate models. We also tested proxy latitudes of maternal residence, gestational diabetes, hypothyroidism and depressive disorder for potential mediating role in offspring gastroschisis development in *winter* versus *summer*. We used the CAUSALMED procedure for this purpose^31^. This method decomposes total effects into several two-, three-, and four-way decompositions. We used causal mediation analysis to decompose the total direct effect of a given risk factor into controlled direct effect, reference interaction, mediated interaction and pure indirect effect^32^. Each potential risk factor was alternatively included in a mediation analysis model as a covariate wherein all other characteristics and factors were controlled. Based on Poisson regression modelling, the value of total effect is equal to the product of mean ratio of the direct and indirect effect.

### Secondary analysis

Gestational diabetes is most often manifest in later pregnancy (after 24 weeks) in response to naturally hormonal changes^32^. It was identified as a confounding in several previous studies of risk factors for gastroschisis^9,33,34^. In our post hoc analysis, we evaluated whether women with gestational diabetes manifested with conception seasonal trend similar to gastroschisis^35^. In addition, we assessed possible mediating role for proxy latitude, gestational diabetes, hypothyroidism and depressive disorder associated with lower gastroschisis prevalence in *summer* months using mediation analysis.

We used omphalocele and chromosomal anomalies (s-Table 1) as negative controls because both are known to be causally distinct from gastroschisis and primarily genetic. We included these two anomalies as negative controls^36^ and applied the same participant inclusion criteria and repeated assessments of the conception seasonal variations, and mediation analysis.

This study was carried out under the surveillance and applied research mandate of the Public Health Agency of Canada, and ethics approval was not required.

## Results

### Characteristics of study cohort

A total of 3,689,855 and 719,861 mother-infant dyads were identified to be conceived in exposure and non-exposure groups, respectively, yielding gastroschisis prevalence rates of 3.40 (95% confidence interval (CI) 3.22–3.60) and 2.21 (95% CI 1.88–2.58) per 10,000 infants. There were moderate temporal variations while no significant differences between the five 2-month periods were identified in the exposure season (χ^2^ = 4.7, *p* = 0.32).

**Figure 2:**
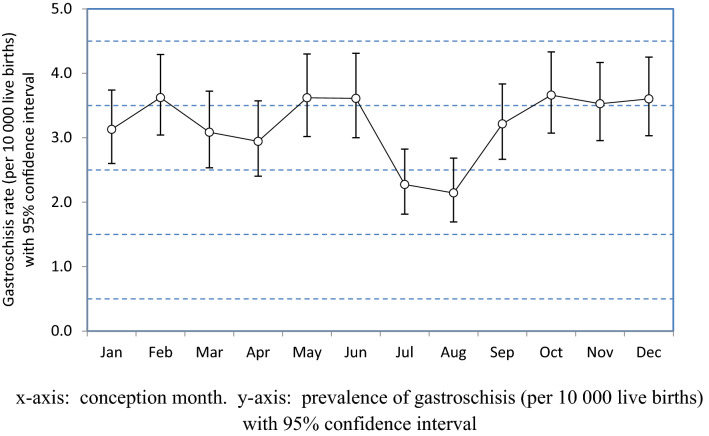
Infant gastroschisis prevalence with 95% CI by conception month in Canada, 2004–2020.

Rates of gastroschisis appeared to vary by month of conception across the 10-month span of the designated *winter* season with a low of 2.94 (95% CI 2.40–3.57) per 10,000 in April to the high values of 3.62 (95% CI 3.02–4.30) in May and 3.66 (95% CI 3.07–4.33) per 10,000 in October. Gastroschisis rates for infants conceived in November (3.53, 95% CI 2.96–4.17) per 10,000 and December (3.60, 95% CI 3.03–4.25) were significantly higher than those conceived in July (2.27, 95% CI 1.81–2.82) and August (2.14, 95% CI 1.67–2.68) per 10,000 infants, respectively (Fig. 2). There were statistically significant differences in geographic region, maternal age at conception, parity, rural or urban residence and substance use disorders. However, the differences in maternal hypothyroidism, depressive disorder, tobacco smoking, obesity, chronic illness and infant sex between the two birth cohorts were statistically non-significant (Table 1).

### Risk or “protective” factors

In the adjusted analysis, compared with the 2-month *summer*, the rate of gastroschisis in infants conceived in *winter* seasons remained higher with an adjusted rate ratio (aRR) 1.55 (95% CI 1.31–1.82). Gastroschisis prevalence increased from 2.4 per 10,000 livebirths in South to 8.8 in far North, with an approximately twofold South-North increase (aRR 1.83, 95% CI 1.34–2.51). Women with hypothyroidism, substance use, depressive disorder, or tobacco smoking showed a significantly higher prevalence rate ratio, while women with gestational diabetes or obesity showed an inverse association with risk of gastroschisis. Specifically, maternal hypothyroidism showed a significant association with gastroschisis (aRR 2.86, 95% CI 1.68–4.85). Substance use disorders remained significantly associated with infant gastroschisis; however, the strength of association was attenuated substantially, from a crude RR: 4.20 (95% CI 3.23–5.46) to an adjusted RR: 2.22 (95% CI 1.70–2.91). The adjusted rate ratio for depressive disorder changed only slightly. In addition, both gestational diabetes and obesity remained negatively associated with infant gastroschisis, although the strength of association reduced considerably after adjustments (Table 2).

**Table 2:** Association of conception season and geographic region with infant gastroschisis.

Variable	Rate ratio & 95% CI^a^	
	Unadjusted	Adjusted
*Exposed*		
* Winter*	1.54 (1.31–1.82)	1.55 (1.31–1.82)
*Unexposed*		
* Summer*	1.00 (reference)	1.00 (reference)
Geographic region		
Far North	3.65 (2.70–4.93)	1.83 (1.34–2.51)
North	2.65 (2.22–3.16)	1.45 (1.20–1.76)
North–South	1.50 (1.34–1.68)	1.31 (1.17–1.47)
South	1.00 (reference)	1.00 (reference)
Hypothyroidism	1.37 (0.81–2.32)	2.86 (1.68–4.85)
Depressive disorder	2.42 (1.34–4.38)	2.23 (1.17–3.90)
Substance use	4.20 (3.22–5.40)	2.22 (1.70–2.91)
Tobacco use	3.74 (2.55–5.47)	1.72 (1.17–2.53)
Gestational diabetes	0.28 (0.18–0.42)	0.45 (0.30–0.67)
Obesity	0.48 (0.26–0.86)	0.54 (0.28–0.91)

^a^Adjustments include all variables in the left column, plus maternal age, chronic illness and infant sex as categorized in Table 1.

### Interaction, two-month comparison and varying rate ratio for the influential factors

All two-month exposure intervals within the *winter* season showed statistically lower adjusted rate ratios of infant gastroschisis compared with *summer*, while there appeared little variation for the overall comparison (i.e., aRR 1.55 vs. cRR 1.54). Interaction terms indicated statistically significant associations (i.e., 6- or sevenfold higher rate ratio) for maternal age (< 24 years vs. 24–44) with specific 2-month *winter* conception seasons and year-around comparisons. Adjusted for covariates, the interaction term showed a significant association with conception season in March–April (aRR 1.96, 95% CI 1.34–2.85), November–December (aRR 1.39, 95% CI 1.01–1.91), and the entire *winter* months (aRR 1.42 (95% CI, 1.19–1.70) compared to *summer* (Table 3).

**Table 3:** Seasonal variations in associations of exposure to specific *winter* months with infant gastroschisis.

Variable	Reference	Regression model for 2-month conception season ^b^					*Winter* versus *Summer*
	July–Aug	Jan–Feb	Mar–April	May–June	Sep–Nov	Nov–Dec	
Risk of infant gastroschisis							
cRR & 95% CI	1.00 (ref)	1.54 (1.25–1.86)	1.36 (1.11–1.68)	1.64 (1.34–2.00)	1.56 (1.28–1.90)	1.61 (1.33–1.96)	1.54 (1.31–1.82)
aRR & 95% CI	1.00 (ref)	1.37 (1.04–1.81)	0.88 (0.63–1.22)	1.54 (1.16–2.04)	1.38 (1.04–1.84)	1.33 (1.01–1.76)	1.55 (1.31–1.82)
Interaction^a^							
cRR & 95% CI	1.00 (ref)	6.17 (5.66–7.51)	7.44 (6.05–9.15)	6.38 (5.22–7.79)	6.72 (5.52–8.17)	6.89 (5.70–8.34)	7.80 (7.02–8.67)
aRR & 95% CI	1.00 (ref)	1.21 (0.88–1.68)	1.96 (1.34–2.85)	1.11 (0.79–1.54)	1.22 (0.88–1.70)	1.39 (1.01–1.91)	1.42 (1.19–1.70)
Depressive disorder							
cRR & 95% CI	1.00 (ref)	2.27 (0.73–7.05)	3.30 (1.23–8.84)	2.15 (0.69–6.70)	3.70 (1.53–8.94)	2.88 (1.08–7.72)	2.42 (1.34–4.38)
aRR & 95% CI	1.00 (ref)	2.09 (0.66–6.59)	3.05 (1.12–8.32)	1.94 (0.61–6.17)	3.24 (1.30–8.08)	2.33 (0.84–6.48)	2.08 (1.13–3.81)
Hypothyroidism							
cRR & 95% CI	1.00 (ref)	2.03 (0.90–4.54)	2.27 (1.01–5.08)	1.31 (0.49–3.51)	1.70 (0.70–4.11)	1.59 (0.66–3.85)	1.37 (0.81–2.32)
aRR & 95% CI	1.00 (ref)	4.25 (1.89–9.56)	5.38 (2.38–12.2)	2.90 (1.08–7.79)	3.76 (1.55–9.14)	3.47 (1.43–8.40)	2.94 (1.77–5.09)
Substance use							
cRR & 95% CI	1.00 (ref)	3.47 (2.21–5.43)	2.63 (1.54–4.49)	2.87 (1.76–4.66)	3.18 (1.99–5.11)	2.96 (1.85–4.75)	3.14 (2.44–4.04)
aRR & 95% CI	1.00 (ref)	2.70 (1.70–4.49)	1.90 (1.10–3.28)	2.36 (1.44–3.88)	2.12 (1.27–3.54)	1.80 (1.06- 3.04)	1.90 (1.47–2.47)
Tobacco smoking							
cRR & 95% CI	1.00 (ref)	4.24 (2.19–8.20)	4.11 (2.04–8.32)	2.80 (1.25–6.28)	3.35 (1.59–7.08)	4.12 (2.13–7.97)	3.74 (2.55–5.47)
aRR & 95% CI	1.00 (ref)	1.90 (0.97–3.73)	1.81 (0.89–3.69)	1.23 (0.55–2.79)	1.49 (0.70–3.17)	1.93 (0.98–3.77)	1.72 (1.17–2.54)
Gestational diabetes							
cRR & 95% CI	1.00 (ref)	0.28 (0.14–0.56)	0.31 (0.16–0.63)	0.29 (0.14–0.58)	0.29 (0.15–0.59)	0.27 (0.14–0.55)	0.28 (0.18–0.42)
aRR & 95% CI	1.00 (ref)	0.50 (0.25–1.02)	0.62 (0.31–1.26)	0.56 (0.28–1.13)	0.57 (0.28–1.14)	0.52 (0.26–1.05)	0.47 (0.31–0.70)
Obesity							
cRR & 95% CI	1.00 (ref)	0.44 (0.14–1.37)	0.49 (0.16–1.52)	0.15 (0.02–1.04)	0.15 (0.02–1.05)	0.42 (0.14–1.32)	0.48 (0.26–0.86)
aRR & 95% CI	1.00 (ref)	0.45 (0.14–1.41)	0.52 (0.17–1.63)	0.15 (0.02–1.07)	0.15 (0.02–1.09)	0.44 (0.14–1.38)	0.50 (0.28–0.91)

^a^Interaction term: Young women (age < 24 years = 1, age 24–44 years = 0) × conception period (individual specific 2-month = 1, July–August = 0).

^b^For each regression model, adjusted also for period of birth, geographic region, maternal age (quadratic term), infant sex, parity, multiple pregnancy, and chronic illness.

Based on the two-month conception seasonal comparisons, rate ratios for hypothyroidism, depressive disorder, substance or tobacco use and gestational diabetes showed significant or substantial changes between the respective crude and fully adjusted models, and between two-month *winter* conception intervals and *summer*. For example, in the comparisons of March–April versus July–August, rate ratios for hypothyroidism and gestational diabetes doubled after adjustments, while adjusted rate ratio for the outcome risk in March–April was attenuated to be non-significant (aRR 0.88, 95% CI 0.63–1.22). Similar changes to rate ratios were also observed for the comparisons between November–December and *summer*. There appeared to be substantial changes in rate ratios for hypothyroidism and gestational diabetes in the 2-month comparisons, indicating that the comparison of entire *winter* versus *summer* underlie the seasonal variations in the outcome measure for exposure *winter* versus non-exposure *summer, w*hile the variations in rate ratios for hypothyroidism and gestational diabetes appeared to primarily vary with the two-month conception intervals (Table 3). It is also notable that tobacco smoking was no longer identified as a risk factor, although substance use remained significantly associated with risk of infant gastroschisis. Taken together, unlike the entire *winter* versus *summer* comparisons, these two-month conception seasonal comparisons seemed to better reflect the risk or protective (instead of confounding) roles for the influential factors of interest in offspring gastroschisis.

### Mediational effects and causal pathway

Maternal depressive disorder, substance use and tobacco smoking showed significant positive total effects of 2.19, 2.18, and 1.69, respectively, on infant gastroschisis, while hypothyroidism was identified to have statistically significant mediational and interactive effects with the first, as well as the third individual risk factors. In particular, depressive disorder showed statistically significant total effects (mean ratio: 2.19 (95% CI 0.87–3.50) on infant gastroschisis while hypothyroidism mediated the relationship (mean ratio: 1.04 (95% CI 1.01–1.07, *p* < 0.001) with 5.3% of total effects (95% CI 1.6–8.9%, *p* < 0.01) and 1.8% as interaction (95% CI 0.8%-2.9%) for risk of infant gastroschisis, suggesting that hypothyroidism impacts offspring gastroschisis development through a mediational path in depressed women. Further, the four-way decomposition of total effect of periconception depression strengthens that pure mediation effect (mean ratio: 0.029; 95% CI 0.015 to 0.044; *p* < 0.0001) may underlie the causal relationship: periconception depression → hypothyroidism (mediator) → grastroschisis (Table 4).

**Table 4:** Maternal hypothyroidism as mediator in the associations of maternal risk or protective factors with infant gastroschisis.

Factor-specific mediation model^a^	Effect	Multivariate mediation analysis^b^	
		Mean ratio & 95% CI	*P* value
Depressive disorder	Natural direct	2.123 (0.843 to 3.404)	0.001
	Natural indirect	1.038 (1.007 to 1.068)	< 0.001
	Total	2.186 (0.869 to 3.503)	0.001
	% mediated	5.25 (1.58 to 8.93)	< 0.01
	% due to interaction	1.81 (0.77 to 2.86)	< 0.001
		*Four-way decomposition*	
	Control direct	1.135 (− 0.158 to 2.428)	> 0.05
	Reference interaction	− 0.011 (− 0.025 to 0.002)	> 0.05
	Mediated Interaction	0.033 (− 0.008 to 0.073)	0.11
	Pure indirect	0.029 (0.015 to 0.044)	0.0001
Substance use disorder	Natural direct	2.182 (1.595 to 2.768)	< 0.001
	Natural indirect	0.998 (0.996 to 0.999)	< 0.01
	Total effect	2.177 (1.592 to 2.762)	< 0.001
	% mediated	− 0.43 (− 0.71 to − 0.15)	< 0.01
	% due to interaction	0.25 (− 0.08 to 0.57)	0.14
Tobacco smoking	Natural direct	1.686 (1.034 to 2.338)	< 0.001
	Natural indirect	1.003 (1.001 to 1.004)	< 0.001
	Total	1.691 (1.037 to 2.344)	< 0.001
	% mediated	0.64 (0.08 to 1.20)	< 0.05
	% interaction	0.56 (0.08 to 1.03)	< 0.05
Gestational diabetes	Natural direct	0.453 (0.269 to 0.637)	< 0.001
	Natural indirect	1.005 (1.003 to 1.008)	< 0.001
	Total	0.469 (0.279 to 0.659)	< 0.001
	% mediated	− 0.42 (− 0.79 to − 0.37)	< 0.001
	% due to interaction	0.65 (0.12 to 1.17)	0.01
Obesity	Natural direct	0.506 (0.205 to 0.808)	0.001
	Natural indirect	1.015 (1.008 to 1.023)	< 0.001
	Total	0.528 (0.214 to 0.843)	0.001
	% mediated	− 1.59 (− 3.66 to 0.49)	0.14
	% due to interaction	1.14 (0.35 to 1.93)	< 0.01

^a^Individual exposures are indicated in each of the mediation models where hypothyroidism is consistently used as mediator, and all other factors in Table 1 are included in the covariate list.

^b^Maternal age, 2-month conception interval, period of birth, latitude and parity, etc. are all included as categorical covariates.

In addition, we also have showed that hypothyroidism may impact offspring gastroschisis through a negative mediational path in pregnant women with substance use disorder (0.998; 95% CI 0.996 to 0.999; *p* < 0.01) or through both mediational and interactive paths in women with tobacco smoking (*p* < 0.05). Gestational diabetes and obesity showed statistically significant negative overall effect (*p* < 0.001) on the risk of infant gastroschisis, respectively, through mediational and/or interactive pathways with maternal hypothyroidism (Table 4).

### Higher infant gastroschisis rate in winter and periconception depression-associated factors

Exposure to *winter* showed significantly higher direct effect (mean ratio: 1.55 (95% CI 1.29–1.80) on risk of offspring gastroschisis that was mediated by latitude (*p* = 0.001). Gestational diabetes in *winter* showed less mediational or “protective” effects (mean ratio: -0.0015 (95% CI − 0.0026 to − 0.0004, *p* < 0.001), while latitude also impacted (i.e., less protective) significantly (mean ratio: − 0.0009 (95% CI − 0.0015 to − 0.0004, *p* = 0.001) on the risk of infant gastroschisis. Specifically, depressive disorder appeared to have additional mediational effect through an interaction of two-month conception seasonal variation with young (< 24 years) pregnant women with a reduced risk of gastroschisis among infants conceived in *summer* (Table 5).

**Table 5:** Additional mediation analyses showing the lower infant gastroschisis rate in summer than winter.

Mediation model/exposure	Mediator	Effect category	Multivariate mediation analysis	
			Mean ratio & 95% CI	*P* value
*Winter* versus *Summer*	Latitude	Natural direct effect	1.546 (1.291 to 1.801)	< 0.001
		Natural indirect effect	0.9994 (0.9990 to 0.9997)	< 0.001
		Excess natural indirect effect	− 0.0009 (− 0.0015 to − 0.0004)	0.001
		Total effect	1.545 (1.290 to 1.800)	< 0.001
	Gestational diabetes	Natural direct Effect	1.548 (1.292 to 1.804)	< 0.001
		Natural indirect effect	0.9997 (0.9995 to 1.0000)	< 0.001
		Excess natural indirect effect	0.9990 (0.9984 to 0.9997)	< 0.05
		Total effect	1.545 (1.290 to 1.799)	< 0.001
	Depressive disorder^a^	Natural direct effect	1.250 (1.003 to 1.496)	< 0.001
		Natural indirect effect	− 0.0015 (− 0.0026 to − 0.0004)	< 0.001
		Excess natural indirect effect	− 3.3 × 10^–4^ (− 6.4 × 10^–4^ to -0.3 × 10^–4^)	< 0.01
		Total effect	1.546 (1.291 to 1.802)	< 0.001

As required by mediation analysis modeling, “treatment” and “mediator” must be numerical, thus, winter (September through June) = 1, summer (i.e., July and August) = 0, and specific mediators (presence = 1, no presence = 0), and proxy latitude (geographic region): far North = 1, North = 0.5, North-to-South = 0.2, South = 0.

In each model, while a factor is used as mediator, all other factors (listed in Table 1) were included in the covariate list.

^a^“Depressive disorder” model includes an “interaction term”- young women (age < 24 years = 1, age 24–44 years = 0) × conception period (individual specified 2-month = 1, July–August = 0). Otherwise, “natural indirect effect” is statistically non-significant.

The rate of maternal depression increased by 50%, from 26.1 per 10,000 in 2004–2007 to 39.0 per 10,000 in 2016–2020, while the fully adjusted rate increased by 19%. In particular, a 1.6-fold increase in depressive disorder was observed in teen women (i.e., age 13–18 years at conception), compared with women aged 24–28 years. More importantly, we observed a 5.5-fold (95% CI 5.2–5.6) association with substance use, 3.1-fold (95% CI 3.8–4.3) risk of hypothyroidism and 2.7-fold increased risk of tobacco smoking in women with depressive disorder. The fully adjusted rate ratio for depressive disorder was significantly lower in *summer* (aRR 0.956, 95% CI 0.915–0.999; *p* < 0.05), highlighting a significantly reduced risk of gastroschisis in infants conceived in *summer* (Table 6).

**Table 6:** Association of maternal characteristics and factors with depressive disorder.

Characteristic	No. of women (% of total) (N = 4,409,716)	Women with depressive disorder	Crude rate ratio (95% confidence intervals)	Adjusted rate ratio^a^ (95% confidence intervals)
Season of conception				
*Winter*	3,689,855	11,833	0.961 (0.920–1.004)	0.956 (0.915–0.999)^b^
*Summer*	719,861	2042	1.00 (reference)	1.00 (reference)
Period of birth				
2004–2007	1,037,160	2706	1.00 (reference)	1.00 (reference)
2008–2011	1,126,078	3510	1.19 (1.14–1.26)	1.11 (1.06–1.17)
2012–2015	1,129,845	3663	1.24 (1.18–1.31)	1.08 (1.02–1.13)
2016–2019	1,116,633	4356	1.50 (1.43–1.57)	1.19 (1.13–1.25)
Maternal age at conception (year)				
13–18	160,936	692	1.59 (1.46–1.72)	1.55 (1.43–1.68)
19–23	617,047	2163	1.29 (1.23–1.36)	1.26 (1.19–1.33)
24–28	1,263,280	3425	1.00 (reference)	1.00 (reference)
29–33	1,475,958	4320	1.08 (1.03–1.13)	1.06 (1.02–1.11)
34–44	892,495	3635	1.50 (1.43–1.57)	1.33 (1.27–1.49)
Substance use				
Yes	44,427	1336	10.2 (9.62–10.8)	5.47 (5.15–5.58)
No	4,345,160	10,288	1.00 (reference)	1.00 (reference)
Tobacco smoking				
Yes	22,834	444	6.19 (5.63–6.80)	2.73 (2.47–3.01)
No	4,386,882	13,791	1.00 (reference)	1.00 (reference)
Gestational diabetes				
Yes	285,975	1213	1.34 (1.27–1.42)	1.23 (1.16–1.30)
No	4,123,741	13,022	1.00 (reference)	1.00 (reference)
Hypothyroidism				
Yes	31,907	573	5.75 (5.29–6.26)	3.06 (3.81–4.33)
No	4,377,809	31,334	1.00 (reference)	1.00 (reference)
Obesity				
Yes	71,342	844	3.83 (3.28–4.11)	2.02 (1.88–2.17)
No	4,338,374	13,391	1.00 (reference)	1.00 (reference)

^a^Adjusted also for parity, rural versus urban residence, geographic region (i.e., latitude), chronic illness, multiple pregnancy and infant sex.

^b^*p* < 0.05.

### Secondary analysis

Gestational diabetes rates according to conception month varied greatly throughout the year, with a significantly lower rate starting in August and returning to an average level in December (s-Fig. 2). Nevertheless, *winter* (i.e., September through June) showed a significantly higher risk for gestational diabetes (95% CI 1.019, 1.009–1.029, *p* = 0.0002) compared to *summer* (July and August) regardless of the covariates (s-Table 2).

We did not observe any statistically significant conception seasonal patterns, decreased or increased prevalence in *summer* compared to other seasons for prevalence of chromosomal anomalies or omphalocele in livebirths (s-Fig. 3, s-Table 3). Mediation analyses for these two negative controls showed that neither residential latitudes nor hypothyroidism had indirect effects on risk of infant omphalocele or chromosomal anomalies (*p* > 0.05). No mechanistic path was identified through the mediation analysis (s-Table 4, s-Table 5).

## Discussion

In this Canada-wide population-based study, we found that infants conceived in July and August were at a significantly lower rate of gastroschisis than those conceived in any other 2-month intervals of the year. This lower rate in *summer* months appeared to be associated with decreased maternal depression at least in part mediated by thyroid function, presumably attributable to the circannual endocrine changes driven by increased exposure to sunlight. Substance use and tobacco smoking are also identified to have direct effects on offspring gastroschisis in the associations of maternal factors with infant gastroschisis. Diagnosed maternal depression around conception showed significant independent effects on offspring gastroschisis development, and our mediation analysis indicates that such effects appear to work through the mechanistic pathway of maternal hypothyroidism. Periconception depression appeared to influence the conception seasonal variations in risk, along with several other associated factors (e.g., residential latitude, young maternal age, and gestational diabetes). Our data suggest that these factors play an interactive role in the development of offspring gastroschisis.

Our findings on these associations and their respective strength and seasonal variation of gastroschisis prevalence with identified risk factors suggest that young women with depressive disorder are at significantly elevated risk of offspring gastroschisis. Exposure to *summer* season appeared to play as an exclusive modifiable factor for the relationship between periconception depression and infant gastroschisis. Our findings provide data supporting the hypothesis that increased periconceptional exposure to *summer* (i.e., longer daytime, lower latitude and more active lifestyle) could protect against gastroschisis during fetal development, in particular, for young women.

There is a substantial body of evidence that shows maternal perinatal mental disorders are associated with an increase in a range of adverse developmental outcomes in offspring. Prenatal stress and associated epigenetic changes in pregnant women with mental disorders may increase the risk of adverse child outcomes^37^. Another example, a recent study from Quebec, Canada reported that maternal depression and stress and anxiety was significantly associated with an increased risk of hypertrophic pyloric stenosis in the newborns^38^.

Two meta-analyses assessed the association between antenatal depression and fetal and neonatal outcomes^10,39^. One reported that studies controlling for women taking antidepressant drugs or smoking generated small (non-significant) odds ratios^39^, whereas the other concluded that the summary relative risk was comparable for depressed women treated and not treated with antidepressants. Antidepressants or smoking can be markers for more severe depression, showing stronger association with antidepressants and mental health disorders^10^. Selective serotonin reuptake inhibitors are widely used for depression and anxiety and have been associated with birth defects in several studies^40,41^. Previous studies reported that use of antidepressants, depression, and stressful pregnancy events were associated with up to 4 times the odds of having a child with gastroschisis^34,42^.

Hypothyroidism is considered a cause of or a strong risk factor for depression or depressive disorders^43^. Associated with clinical depression, maternal hypothyroidism is relatively common during pregnancy, with an overall prevalence of 0.61% for overt hypothyroidism and 5.1% for subclinical hypothyroidism^44^. We observed 0.72% of hypothyroidism in this data (Table 1). Some past studies have identified maternal hypothyroidism as a possible risk factor for birth defects^45,46^. Light exposure has been associated with seasonal fluctuation of thyroid function related to circulating hormones, and external stimuli such as light and warm temperature can influence thyroid function^11^. Normal maternal thyroid function is essential for optimal pregnancy outcomes, especially during the early gestational period. Firstly, temperature variation is a critical stimulus to the central regulation of hypothalamus-pituitary-thyroid (HPT) axis via changes in secretion of thyroid-stimulating hormone (TSH)^47^. One systematic review and meta-analysis found that individuals with hypothyroidism had significantly lower vitamin D levels compared to healthy people^48^. Secondly, light is associated with seasonal fluctuations in thyroid function. The alteration in thyroid hormone regulation could also be a part of metabolic adaptation to seasonal climate changes^11^. Thirdly, hypothyroidism has been causally associated with decreased sex hormone concentrations such as sex hormone binding globulin (SHBG) and free androgen index (FAI) in women^13^. In addition, increased sunlight and vitamin D_3_ exposure in summer was found to be positively associated with steroid hormone production of sex hormones^18^. Exposure to winter sunlight in Northern Canada does not promote previtamin D_3_ synthesis in human skin^49^.

Based on the findings of our study, we propose the following hypothetical mechanistic pathway by which maternal hypothyroidism could be a key causal factor in the occurrence of many cases of gastroschisis. Thyroid hormone is essential for normal embryonic and fetal development. Maternal thyroid hormone is important for the well-being of the embryo/fetus throughout pregnancy but is the sole source prior to onset of thyroid hormone synthesis and secretion by the fetal thyroid tissue beginning at approximately 16 weeks of pregnancy^50^. Prior to that time, multiple major embryonic events unfold and are solely dependent upon that maternal source of thyroid hormone. While it is well-known that normal neurocognitive development depends upon normal thyroid exposure of developing neural tissues^51^, it is also well understood that the process of angiogenesis including developmental neovascularization is also thyroid hormone dependent^52^. Specifically, the alphaVbeta3 integrin is a key mediator of angiogenesis in adult tissues, tumors and during development, and is directly regulated by a non-genomic thyroid hormone receptor within alphaVbeta3 integrin^53^^,^^54^. Accordingly, absent or reduced thyroid hormone signaling compromises angiogenesis. Since vascular insufficiency or compromise of the relevant abdominal wall vessels is one of the favored hypothetical mechanisms by which gastroschisis occurs, reduced or absent angiogenesis could be a key factor in the occurrence of gastroschisis. In brief, maternal thyroid hormone is the sole source for the embryo, and that maternal thyroid hormone acts locally on the plasma membrane receptor located within alphaVbeta3 integrin to stimulate angiogenesis in the embryo. If maternal thyroid hormone supplied to the embryo is modestly, moderately or severely reduced, the angiogenesis that is essential for normal development of the abdominal wall of the embryo/fetus would be compromised.

To our knowledge, this is the first study to report the role of light and presumptive active lifestyle exposure associated with maternal depression, hypothyroidism, and potential mood/mental changes in the causality of fetal gastroschisis development. The strengths of our study include use of data from a vast geography with differential latitude/climate (e.g., far North) along with distinctive seasonal variation. Information on postal codes for maternal residence is complete and accurate and has been used in the past to account for differences in pregnancy-related endpoints such as prenatal screening or pregnancy termination. Our secondary analysis of gestational diabetes by conception season may also elucidate the inverse associations of gastroschisis with gestational diabetes observed in previous studies^9,34,35^.

However, several limitations must be noted. Firstly, some risk factors that are associated with gastroschisis may be tested after the occurrence of a congenital anomaly or other adverse pregnancy outcome. As antenatal data could not be available for our analysis, subclinical hypothyroidism and depression cases could have been missed in the childbirth hospitalization data. The clinical manifestations may represent a maternal persistent condition. Secondly, misclassification or lack of diagnosis may occur, though probably not in a differential manner, and thus it is not plausible that the coding of hypothyroidism, clinical depression or other conditions would be different for women who conceived in different months. While this potential misclassification may have underestimated the outcome rates, the rate ratio would still be unchanged. Thirdly, while fetal death or stillbirths due to gastroschisis have become more uncommon in Canada, absence of the data may impact the seasonal prevalence and regional variation. In a previous study including stillbirths or more severe or fatal gastroschisis, we reported an increased gastroschisis risk for maternal hypothyroidism but not for depression in Canadian births^9^. We speculate that maternal depression may be more likely to be associated with a somewhat less severe spectrum of gastroschisis in liveborn neonates. Future studies are needed to confirm our observation.

Our study uses data from a vast geography with differential latitudes (i.e., sunlight or active lifestyle exposure), and demonstrates a distinctive seasonal variation. Information on postal codes for maternal residence is complete and accurate and has been successfully used to define residential location (i.e., rural or urban areas) in the past^28,55^. Nevertheless, remaining variations in socioeconomic status, education, diet, ethnicity, vitamin D supplementation, etc., could have confounded our analysis when comparing geographic regions of maternal residence.

In conclusion, given the seasonal and geographic variations in gastroschisis occurrence in Canada and many other parts of the world, particularly in the Northern hemisphere, we observed that northern regions with less daylight length and presumably less active lifestyle might be associated with an increased risk of maternal depression and associated hypothyroidism that is linked to offspring gastroschisis. Our study has demonstrated that conceptions occurring in shorter photoperiod months or higher latitudes are at increased risk of infant gastroschisis compared to longer light months of *summer* and/or lower latitudes in which maternal hypothyroidism may play a significant mediating role in depressed women. Our findings that infant gastroschisis is associated with seasonal and regional factors indicative of low light exposure suggest that seasonal changes in other hormones, such as sex hormones, might also play a role in the etiology of fetal gastroschisis development. Further studies are warranted to confirm this suggestion in other areas of the world, and to identify any biological mechanisms that may link variations in sex hormones with maternal mood (e.g., depression) and hypothyroidism, and in turn with offspring gastroschisis development.

## Supplementary Information

Supplementary Information.

## Data Availability

All data files are available through the Canadian Institute for Health Information (https://www.cihi.ca/en/data-andstandards/access-data).
