# Supplementary material for: Association of maternal depression and hypothyroidism with infant gastroschisis: a population-based cohort study in Canada
Source: Sci Rep. 2023 May 9;13:7540. doi: 10.1038/s41598-023-34090-2 (PMC10170067; doi:10.1038/s41598-023-34090-2)
Supplement: Supplementary file 1 — Supplementary Information. [file 41598_2023_34090_MOESM1_ESM.docx]

s-Figure 1. Flow Chart Detailing Infant Gastroschisis Causality Study Cohort

Live births from April 1, 2004 to March 31, 2020

N = 4 435 732 (1419)^a^

**Excluded:**

12 601 Missing or unknown or <22 or >42 weeks of gestation, birthweight or birth month or mother-newborn linkage number

Eligible mother-liveborn dyads

4 423 131

**Excluded:**

3832 Maternal age <13 or >44 years at conception

9583 non-Canadian or unknown residence (4)^a^

Mother-liveborn dyads

N = 4 409 716 (1415)^a^

##

**Conception in non-exposure season**

Total (%) Case (%)

Jul - Aug 719 861 (16.3) 159 (11.3)

**Conception in exposure season**

Total (%) Case (%)

Jan-Feb 765 147 (17.4) 258 (18.2)

Mar-April 700 015 (15.9) 211 (14.9)

May-June 691 528 (15.7) 250 (17.7)

Sep-Oct 750 468 (17.0) 258 (18.2)

Nov-Dec 782 697 (17.7) 279 (19.7)

Data source: Canadian Information for Health Institute Birth Database

^a^ Denotes number of gastroschisis case in bracket.

s-Table 1

The Canadian Version of the 10^th^ Revision of the International Classification of Disease

(ICD-10 CA) Codes

| **Definition** | ICD-10 CA codes |
| --- | --- |
| **Newborn outcome** |  |
| Gastroschisis | Q79.3 |
| Omphalocele | Q79.2 |
| Down syndrome | Q90 |
| Chromosomal anomalies | Q91-Q92, Q96-Q99 |
| **Maternal characteristic/covariate** |  |
| Gestational diabetes mellitus | O24.0-O24.4, O24.7-O24.9 |
| Problematic tobacco use | F17, T51.2, Z50.8, Z71.6, Z72.0 |
| Substance use disorder |  |
| Problematic use of alcohol | F10, K70,Y15, X45, X65, K29.2, K86.0, K86.9, T51.0, T51.8, T51.9, R78.0,O35.4, Z50.2, Z50.8, Z71.4, Z72.1, Z86.40 |
| Opiate or opioids | F11, R78.1, P96.1 |
| Cannabinoids | F12, T40.7 |
| Cocaine | F14, R82.5, R78.2, T40.5 |
| Other specified substances | F13, F15, F16, F18, F19 |
| Miscellaneous | O35.5, R78.3, R78.4, R78.5, X42, X62, Y12-Y14, Z50.3, Z71.5, Z72.2, Z86.4 |
| Subclinical iodine-deficiency | E02, E03 |
| hypothyroidism /other hypothyroidism |  |
| Depressive episode/recurrent depressive | F32, F33 |
| disorder |  |
| Obesity | E66 |
| Chronic illness/condition |  |
| Pregestational diabetes mellitus type 1 | E10 |
| Pregestational diabetes mellitus type 2 | E11 |
| Hyperthyroidism | E05, E06 |
| Bipolar disorder, anxiety, and other specific mental disorders | F20, F22, F23, F25, F28-F31, F34, F40, F41 |
| Epilepsy | G40 |
| Lupus | L93.0-L93.2, M013, M32 |
| Migraine | G43 |

s-Figure 2. Gestational Diabetes Rate (per 100 Mothers) according to Conception Month in Canada, 2004 – 2020

s-Table 2. Seasonal Variations in Gestational Diabetes According to Conception in

*Winter* months versus *Summer*

| Exposure  /conception month | Rate  (%) | Crude rate ratio  & 95% CI | P value | Adjusted rate ratio & 95% CI ^a^ | P value |
| --- | --- | --- | --- | --- | --- |
| **Two-season comparison** |  |  |  |  |  |
| *Winter(Sept to December)* | 6.51 | 1.021 (1.011 to 1.031) | <0.0001 | 1.019 (1.009 – 1.029) | 0.0002 |
| *Summer* (July and August) | 6.37 | 1.000 (reference) |  | 1.000 (reference) |  |
| **Two-month comparison** |  |  |  |  |  |
| January to February | 6.67 | 1.046 (1.033 to 1.059) | <0.0001 | 1.045 (1.032 - 1.059) | <0.0001 |
| March to April | 6.81 | 1.069 (1.055 to 1.083) | <0.0001 | 1.063 (1.050 – 1.077) | <0.0001 |
| May to June | 6.64 | 1.041 (1.028 to 1.055) | <0.0001 | 1.035 (1.022 – 1.049) | <0.0001 |
| July to August | 6.37 | 1.000 (reference) |  | 1.000 (reference) |  |
| September to October | 6.09 | 0.956 (0.943 – 0.968) | <0.0001 | 0.958 (0.946 - 0.971) | <0.0001 |
| November to December | 6.36 | 0.998 (0.986 - 1.011) | 0.76 | 0.998 (0.986 – 1.011) | 0.80 |

^a^ Poisson regression adjusting for all covariates in Table 1

s-Figure 3. Prevalence of Specific Chromosomal Anomalies

with 95% CI by Conception Month in Canada (excluding province of Quebec), 2004-2020

x-axis: conception month. y-axis: prevalence of selected chromosomal anomalies (per 10 000 live births) with 95% confidence interval

Note: Down syndrome cases are not included as some cases may be associated with gestational diabetes.

s-Table 3. No seasonal variation in infant prevalence of omphalocele and chromosomal anomalies among mother-infant dyads, Canada, 2004 through 2020

| Month of Conception | Number of mother (%) | Omphalocele | | Chromosomal anomalies* | |
| --- | --- | --- | --- | --- | --- |
|  |  | Number (%) | 95% Confidence interval | Number (%) | 95% Confidence interval |
| *Exposure* |  |  |  |  |  |
| Jan – Feb | 765 147 (17.4) | 115 (18.3) | 1.11 (0.85 – 1.46) | 324 (17.8) | 1.07 (0.92 – 1.26) |
| Mar – Apr | 700 015 (15.9) | 93 (14.8) | 0.99 (0.74 – 1.31) | 293 (16.1) | 1.06 (0.90 – 1.25) |
| May – Jun | 691 528 (15.7) | 120 (19.1) | 1.26 (0.97 – 1.65) | 306 (16.7) | 1.12 (0.95 – 1.32) |
| Sep - Oct | 750 468 (17.0) | 102 (16.2) | 1.00 (0.76 – 1.32) | 308 (17.3) | 1.04 (0.89 – 1.22) |
| Nov – Dec | 782 697 (17.7) | 101 (16.1) | 0.96 (0.73 – 1.26) | 300 (16.5) | 0.97 (0.83 - 1.14) |
| *Non-Exposure* |  |  |  |  |  |
| Jul - Aug | 719 861 (16.3) | 98 (15.6) | 1.00 | 284 (15.6) | 1.00 |
| Total | 4 409 716 (100.0) | 629 (100.0) |  | 1815 (100.0) |  |

*Down’s syndrome cases are not included as some cases may be associated with gestational diabetes.

s-Table 4. Mediation analysis showing no relation between maternal hypothyroidism and offspring omphalocele or selected chromosomal anomalies*

| Multivariate mediation analysis | Omphalocele | | Chromosomal anomalies* | |
| --- | --- | --- | --- | --- |
|  | Adjusted mean ratio & 95% Confidence interval | | Adjusted mean ratio & 95% Confidence interval | |
| Natural Direct effect | 1.059 (0.831 to 1.288) | <0.0001 | 1.049 (0.917 to 1.183) | <0.0001 |
| Natural Indirect effect | 1.000 (1.000 to 1.000) | <0.0001 | 1.000 (1.000 to 1.000) | <0.0001 |
| Total effect | 1.059 (0.831 to 1.288) | <0.0001 | 1.050 (0.916 to 1.182) | <0.0001 |
| Excess total effect | 0.060 (-0.170 to 0.290) | 0.61 | 0.050 (-0.083 to 0.183) | 0.46 |
| Excess direct effect | 0.59 (-0.17 to 0.29) | 0.61 | 0.050 (-0.083 to 0.183) | 0.46 |
| Excess indirect effect | 1.2 x10-6 (-0.3 x 10-4 to 0.3 x 10-4) | 0.94 | -5.1x10-7 (-0.1 x 10-4 to 0.1 x 10-4) | 0.94 |

*Down’s syndrome cases are not included as some cases may be associated with gestational diabetes.

s-Table 5. Mediation analysis showing no relation between gestational diabetes and offspring omphalocele or selected chromosomal anomalies*

| Multivariate mediation analysis | Omphalocele | | Chromosomal anomalies * | |
| --- | --- | --- | --- | --- |
|  | Adjusted mean ratio & 95% Confidence interval | | Adjusted mean ratio & 95% Confidence interval | |
| Natural Direct effect | 1.055 (0.828 to 1.283) | <0.0001 | 1.049 (0.916 to 1.182) | <0.0001 |
| Natural Indirect effect | 1.0004 (0.9998 to 1.0001) | <0.0001 | 1.0002 (0.9999 to 1.0006) | <0.0001 |
| Total effect | 1.056 (0.828 to 1.283) | <0.0001 | 1.049 (0.916 to 1.182) | <0.0001 |
| Excess direct effect | 0.055 (-0.172 to 0.283) | 0.63 | 0.049 (-0.0837 to 0.1822) | 0.47 |
| Excess indirect effect | 0.0041 (-0.00023 to 0.0011) | 0.21 | 0.00024 (-0.0001 to 0.0006) | 0.20 |
| Excess total effect | 0.056 (-0.174 to 0.279) | 0.65 | 0.0485 (-0.0845 to 0.1815) | 0.48 |

*Down’s syndrome cases are not included as some cases may be associated with gestational diabetes.
